# Prevalence of metabolic syndrome among adolescents in India: a population-based study

**DOI:** 10.1186/s12902-022-01163-8

**Published:** 2022-10-24

**Authors:** Sowmya Ramesh, Ransi Ann Abraham, Avina Sarna, Harshpal S. Sachdev, Akash Porwal, Nizamuddin Khan, Rajib Acharya, Praween K. Agrawal, Sana Ashraf, Lakshmi Ramakrishnan

**Affiliations:** 1 Population Council, Zone 5A, Ground Floor India Habitat Centre, Lodi Road, New Delhi, Delhi, 110003 India; 2 Cardiac Biochemistry, All India Institute of Medical Sciences, Delhi, India; 3 Sitaram Bhartia Institute of Science and Research, New Delhi, India; 4 IPE Global Limited, New Delhi, India

**Keywords:** Adolescents, Metabolic syndrome, CNNS, India

## Abstract

**Background:**

In India, the prevalence of overweight among adolescents is on the rise, setting the stage for an increase in metabolic syndrome (MS). This paper presents the national prevalence of MS in adolescents in India.

**Methods:**

A nationally representative data of adolescents (10–19 years) from the Comprehensive National Nutrition Survey was used. MS was defined based on the NCEP–ATP III criteria for adolescents. Bivariate analysis was used to report socio-demographic differentials in prevalence and to assess interstate variability. Multivariate logistic regression model was constructed to measure the association between socio-demographic characteristics and prevalence of MS. Census data from 2011 was projected to 2017 to calculate burden.

**Results:**

The prevalence of MS was 5.2% among adolescents. 11.9%, 15.4%, 26.0%, 31.9% and 3.7% had central obesity, high blood pressure, hypertriglyceridemia, low HDL-cholesterol and high fasting glucose, respectively. The prevalence was higher among males (5.7% vs. 4.7%, adjusted odds ratio (AOR): 1.3, 95% confidence interval [CI]: 1.0, 1.6), those residing in urban areas (7.9% vs 4.2%, AOR: 1.4, 95% CI: 1.1, 1.8), and from wealthier households as compared to their counterparts (8.3% vs. 2.4%, AOR: 3.4, 95% CI: 2.1, 5.5). There was wide interstate variability in the prevalence of MS (0.5% – 16.5%). In 2017, 14.2 million adolescents had MS in India.

**Conclusions:**

The prevalence of MS among adolescents in India is low and clustered in urban areas and richer households. Early prevention interventions promoting a healthy lifestyle, especially in high prevalence areas, are needed to keep MS from becoming a public health issue.

## Background

The prevalence of obesity among adolescents has increased over the last decade. In the Indian context, a similar trend can be witnessed among adolescents. According to National Family Health Survey – 4 (NFHS-4) conducted in 2015–16, the prevalence of overweight or obesity among adolescents aged 15 to 19 years was reported to be 4.2%, a significant increase from the previous round [1, 2]. As well, a systematic review conducted among adolescents aged 10 to 18 years in 2016 in India indicated increasing rates of overweight and obesity [3]. The study highlighted that the prevalence was not increasing just among adolescents from the higher socio-economic strata but also among the lower income groups where undernutrition continues to be a major public health issue. [3]. A growing proportion of the adolescent population with overweight and obesity sets the stage for a potential increase in metabolic syndrome (MS) as global research indicates a connection between the two [4].

MS is a cluster of conditions, including central adiposity, dyslipidemia, impaired glucose tolerance and hypertension, that occur together [5, 6]. Several studies conducted across the globe such as the Framingham Offspring Study, Kuopio Ischemic Heart Disease Study, NHANES Mortality Study, have reported a significant increase in mortality and cardiovascular complications among adults with MS [7–12]. Studies indicate that children and adolescents with MS have an increased risk of MS as adults, increasing their risk of developing type 2 diabetes mellitus and cardiovascular disease later in life [13–15]. Due to this, increasing efforts have been directed towards understanding MS’ patho-physiology, risk factors and levels in different countries, to identify strategies to manage MS at an early age.

Different criteria have been used to define MS. Reaven and team first described MS in 1988, as a cluster of changes associated with resistance to insulin-mediated glucose uptake, including increase in plasma triglyceride, decrease in high density lipoprotein-cholesterol concentration and high blood pressure [16]. Later, the World Health Organization (WHO) [17], National Cholesterol Education Program and Adult Treatment Panel III (NCEP-ATP III) [18, 19], American Association of Clinical Endocrinologists (AACE) [20] and International Diabetes Federation published their criteria for MS for adolescents [21]. Since 1988, more than 40 different definitions of MS have been in use [22]. The prevalence of MS varies by definition. A systematic review among adolescents in low to medium-income countries reported a prevalence ranging from 4.2%–15.4% among studies that used the NCEP-ATP III criteria; and 4.5%–38.7% in studies where the WHO criteria was used [23]. Researchers who used the same data to estimate the prevalence of MS using different definition reported different prevalence estimates, ranging from 4.2%‍ to 9.2% [24]. Recently, few small-scale studies across India estimated the prevalence of MS among adolescents [4, 25–28]. In Delhi, the reported prevalence was 6.5% among children aged 6–18 years belonging to higher socio-economic strata [25]. Similarly, in Jammu and Kashmir the reported prevalence was 2.6% among adolescents [4]. Yet another study conducted among urban Indian adolescents reported a prevalence of 4.3% and 3.0% using NCEP ATP III and IDF criteria, respectively [27]. Another study identified MS in 4.3% of Asian Indian adolescents [28].

Estimates on the prevalence of MS among adolescents in India are derived from small scale or regional studies; however, no estimates available at the national level. This study presents the national and state prevalence and burden of MS in adolescents aged 10–19 years and the associated socio-demographic differentials.

## Methods

### Design, setting and sample

**Fig. 1:**
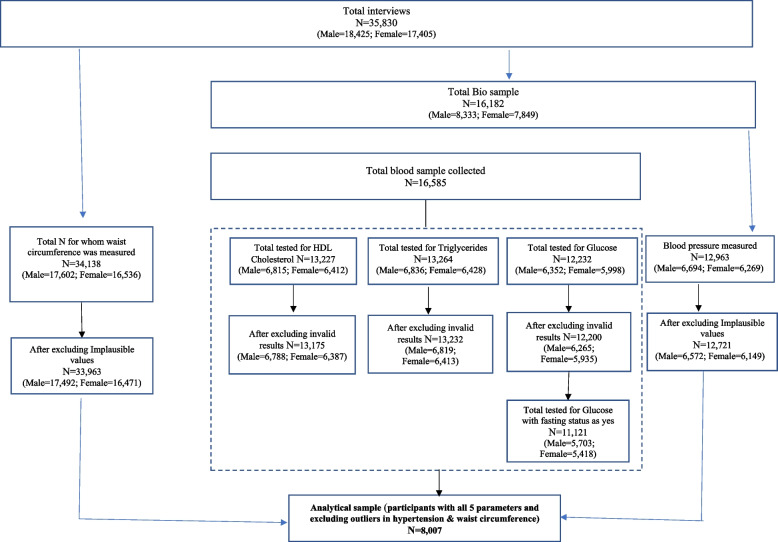
Sample size of various biochemical and anthropometric parameters, CNNS, 2016–18

Data for this study was drawn from the Comprehensive National Nutrition Survey (CNNS), a nationally representative sample of children and adolescents (0–19 years), conducted across all 29 states of India and the capital Delhi in 2016–18. CNNS used a multi-stage, stratified, probability proportion to size cluster sampling to recruit participants. This study used data of adolescents aged 10–19 years. Adolescents with any current illness, chronic disease or physical deformity were excluded from participation in CNNS. Half of all adolescents from whom anthropometric measurements were taken were selected by systematic random sampling and were invited to enroll for biological sampling. Out of these, participants with data for waist circumference, blood pressure, triglycerides, HDL cholesterol and glucose were included in the analytical sample (*N* = 8,007). Details of the survey design and sampling methodology are published elsewhere [29]. Figure 1 presents the details of the sample size for various parameters and the final analytical sample. In CNNS, the sample size for biochemical components was calculated for varying level of prevalence and coefficient of variation (CV), considering minimum response rate of 75% and design effect as 1.5. The minimum sample needed for MS study, with prior prevalence estimates, were 1700 for 15% CV and 950 for 20% CV. The analytical sample for this study was 8,007, which was sufficient for estimating the prevalence of MS.

#### Blood sample collection and quality control

Trained phlebotomists collected blood samples from adolescents and accredited private reference laboratories in Mumbai, Delhi and Kolkata were utilized to conduct all tests included in the study. Rigorous quality control measures and monitoring systems were established for sample collection, transportation and testing. The laboratories had their own internal Quality Control (QC) procedures of the reference laboratory. For external quality assurance, laboratories participated in the BIORAD and US Centre for Disease Control external quality assurance scheme. A subset of samples (5%) was sent to other laboratories (All India Institute of Medical Sciences [AIIMS], Delhi, and the National Institute of Nutrition [NIN], Hyderabad) on a monthly basis for comparison testing for quality control. Technical Advisory Group (TAG) members, constituted for CNNS and experts from NIN, AIIMS and CDSA made regular visits to the field and the laboratories to ensure that the Standard Operating Procedures were followed for collection, transportation and analysis of biological samples.

#### Anthropometric data collection and quality control

Anthropometric data was collected by trained and standardized female health investigators and a three-tier monitoring system was established for quality control of anthropometric data collection. Level 1 monitoring involved an internal quality control observer within the field team to oversee anthropometric equipment calibration and anthropometric measurements. Level 2 comprised, monitoring and supervision undertaken by an external three-member data quality assurance team in each state. The three-member team observed and re-measured at least three respondents in each PSU they visited. Level 3 involved monitoring by Postgraduate Institute for Medical Education and Research (PGIMER) Chandigarh, UNICEF and Population Council to ensure that the measurements were taken as per protocol. Further details of the quality control protocol are available elsewhere [29].

### Measures

#### Biological and anthropometric measures

HDL cholesterol was assessed by spectrophotometry and direct measure polyethylene glycol modified cholesterol oxidase methods; triglycerides were estimated by spectrophotometry and enzymatic endpoint method and glucose was estimated using spectrophotometry, Hexokinase method [30, 31]. Waist circumference was measured in centimeters (to the nearest 0.1 cm), using a non-elastic fiberglass measuring tape, at the midpoint between the lowest rib and the iliac crest in the mid-axillary line at the end of normal expiration [32]. A mean of two readings was recorded. Blood pressure was measured using an automated device. Three blood pressure readings were taken with a gap of at least two minutes and the mean of the last two readings was used for the analysis. Detailed methodology for biological sample collection and laboratory analysis methods used in CNNS is available elsewhere [29].

#### Metabolic syndrome

The criteria considered for MS in this study was based on the NCEP ATP III criteria modified for age, defined as the coexistence of at least three of following five risk factors: high waist circumference (WC) defined as WC ≥ 90th percentile for age and sex, elevated arterial pressure defined as systolic blood pressure (SBP) and/or diastolic blood pressure (DBP) ≥ 90th percentile for age and sex, impaired fasting glucose defined as fasting blood glucose of 110 mg/dL or more, hypertriglyceridemia defined as triglycerides of 110 mg/dL or more and Low HDL defined as HDL cholesterol ≤ 40 mg/dL [19, 33].

#### Background characteristics

Background characteristics included in this study were sex (male, female), age (10–12 years, 13–15 years, 16–19 years), current schooling status of adolescents (Yes, No), area of residence (rural, urban), religion (Hindu, Muslim, Sikh, Christians and Others), caste (Scheduled Caste, Scheduled Tribe, Other Backward Class and Others), and household wealth (wealth index: poorest, poor, middle, rich, richest). Wealth index was created by giving scores that were derived using principal component analysis to households based on the number and kinds of consumer goods they owned and their housing characteristics [2]. Following this step, the household score was assigned to each usual household member, ranked based on their score, and then divided by the distribution into five equal categories, each with 20 percent of the population.

#### Unhealthy diet

In addition, an attempt was made to assess the association between the prevalence of MS and adolescents’ consumption of an unhealthy diet. An unhealthy diet included daily consumption of either fried Indian foods such as *pooris*, *pakoras*, *vadas*, *samosas*, *tikkis* etc.; junk foods such as burgers, pizzas, pastas, instant noodles etc. or sweets such as Indian sweets, pastries/cakes, donuts or aerated drinks.

#### Statistical analysis

Appropriate sampling weights were used to account for non-response rates and differential probabilities for selection of participants across states. Descriptive statistics were generated for the anthropometric and biochemical data. Mean and standard deviation (SD) are presented. The summary statistics for WC, SBP, DBP, fasting glucose levels, triglyceride and HDL cholesterol level were compared between males and females using t-test and Wilcoxon rank sum test. The prevalence of individual risk factors of MS with 95% confidence intervals (CI) was estimated at the national level. The prevalence of MS with 95% CI was estimated at the national, as well as for individual states. Univariate analysis was used to present the prevalence of coexistence of different MS risk factors. The burden of MS in different states and at the national level was estimated using population projections for the year 2017, based on Government of India, Census 2001 and 2011 data. Bivariate analysis was used to report socio-demographic differentials in prevalence and to assess interstate variability. Multivariate logistic regression model was constructed to measure the association between socio-demographic characteristics and prevalence of MS. Adjusted odds ratios (AOR) (adjusted for sex, age, current schooling status of adolescents, area of residence, religion, caste, and household wealth) and corresponding 95% CI are presented in the tables. Stata version 16.0 (College Station, TX, USA) was used for all analyses.

#### Ethical considerations

The research was performed in accordance with the Declaration of Helsinki. Ethical approval for conducting CNNS was received from the Ethics Committee of the Postgraduate Institute for Medical Education and Research in Chandigarh, India, and the Institutional Review Board of the Population Council in New York. A comprehensive consent process was adopted. All aspects of the study were conveyed to the participants prior to taking consent, including the purpose of the study, their right to discontinue anytime during the study without any penalty, they were assured of the confidentiality of data, they were informed that there will be no monetary benefits of participation in the study and they will not experience any serious risks due to participation in the study. For adolescents aged 10–17 years, written informed consent was obtained from the caregivers and written assent was obtained from the adolescents. Adolescents aged 18–19 years provided their own consent.

## Results

Overall, the mean WC, SBP, DBP, glucose level, triglyceride level and HDL cholesterol level among adolescents was 63.0 cm, 112.1 mm of Hg, 71.8 mm of Hg, 89.4 mg/dL, 94.0, mg/d and 46.0 mg/dL respectively (Table 1). Male adolescents had a significantly higher WC and fasting blood glucose level, compared to females. Whereas, females had significantly higher values for triglycerides and HDL-Cholesterols, compared to males. It was found that the SBP was higher among males, whereas DBP was higher among females.

**Table 1:** Descriptive statistics of key risk factors of metabolic syndrome among adolescents, India, CNNS 2016–18

Variable	All (*N* = 8,007)		Male (*N* = 4,067)		Female (*N* = 3,940)	
	**Mean**	**Standard Deviation**	**Mean**	**Standard Deviation**	**Mean**	**Standard Deviation**
**Anthropometric Measurements**						
Waist circumference (cm)^**c**^	63.0	8.3	63.7	8.6	62.3	7.8
**Blood Pressure and Biochemical Measurements**						
Systolic blood pressure (mmHg)^**c**^	112.1	10.6	112.6	10.6	111.7	10.6
Diastolic blood pressure (mmHg)^**a**^	71.8	9.1	71.6	9.1	72.1	9.1
Glucose ( mg/dL)^**b**^	89.4	10.9	89.7	11.3	89.0	10.4
Triglycerides (mg/dL)^**c**^	94.0	50.1	90.1	46.0	98.1	53.6
HDL (mg/dL)^**c**^	46.0	10.4	45.4	10.6	46.6	10.2

^a^*p* < 0.05

^b^*p* < 0.01

^c^*P* < 0.001

The prevalence of each component of MS is presented in Table 2. Low HDL cholesterol was the most prevalent risk factor of MS among adolescents (31.9%), followed by hypertriglyceridemia (26%), hypertension (15%) and central adiposity (12%). Impaired glucose was the least prevalent risk factor (3.7%). More than three-fifth (62%) of adolescents were found to have at least one risk factor of MS (Table 3). Higher proportion of males had at least one risk factor of MS compared to females (63% vs. 60%, *p*-value < 0.01). Less than one percent of adolescents had four or five risk factors of MS.

**Table 2:** Prevalence of individual risk factors of metabolic syndrome among adolescents, India, CNNS 2016–18

Risk factors	All			Male			Female		
	**Prevalence (%)**	**95% CI**	**Total N**	**Prevalence (%)**	**95% CI**	**N**	**Prevalence (%)**	**95% CI**	**N**
Central obesity	11.9	10.1, 13.8	8,007	12.2	9.9, 15.0	4,067	11.4	9.7, 13.5	3,940
Hypertension	15.4	13.4, 17.7	8,007	15.9	13.2, 18.9	4,067	15.0	12.9, 17.4	3,940
Hypertriglyceridemia	26.0	23.3, 28.9	8,007	23.3	20.0, 26.9	4,067	28.7	25.1, 32.6	3,940
Low HDL	31.9	29.1, 34.9	8,007	34.7	30.9, 38.8	4,067	29.0	25.8, 32.4	3,940
Impaired glucose	3.7	3.0, 4.5	8,007	4.4	3.3, 5.7	4,067	3.1	2.3, 4.0	3,940

Note: prevalence presented here is for the sub-sample of adolescents for whom the values for all parameters were available

**Table 3:** Prevalence of number of risk factors of metabolic syndrome among adolescents, India, CNNS 2016–18

No. of risk factors of Metabolic syndrome	All		Male		Female	
	**%**	**N**	**%**	**N**	**%**	**N**
0	38.5	3,006	37.3	1,532	39.7	1,474
1	39.8	3,014	41.2	1,542	38.4	1,472
2	16.5	1,465	15.8	725	17.2	740
3	4.7	449	5.0	229	4.4	220
4	0.5	68	0.7	37	0.3	31
5	0.0	5	0.0	2	0.1	3

Overall, the prevalence of MS was 5.2% among adolescents (Table 4). The prevalence was higher among males compared to females (5.7% vs. 4.7%, AOR: 1.3, 95% CI: 1.0, 1.6). Adolescents aged 13–15 years had higher odds of having MS as compared to those in the age group of 10–12 years (10–12 years vs. 13–15 years: 4.2% vs. 6.4%, AOR: 1.5, 95% CI: 1.1, 2.0). Adolescents who reported being in school at the time of the survey had lower odds of having MS, compared to those not in school (4.9% vs 6.1%, AOR: 0.6, 95% CI: 0.5, 0.8). A significant association was observed between the prevalence of MS and area of residence, with higher proportion of adolescents residing in urban areas having MS, compared to those in rural areas (7.9% vs. 4.2%, AOR: 1.4, 95% CI: 1.1, 1.8). Further, in terms of household wealth, significantly higher proportion of adolescents belonging to wealthier households had a higher chance of getting diagnosed with MS as compared to those from the poorest wealth quintile (8.3% vs. 2.4%, AOR: 3.4, 95% CI: 2.1, 5.5).

**Table 4:** Prevalence of the metabolic syndrome among adolescents by demographic characteristics and diet, India, CNNS 2016–18

Characteristics	Prevalence	95% CI	AOR (95% CI)	*p*-value	Total N
**Sex**					
Male	5.7	3.9, 8.2	1.3 (1.0, 1.6)	0.048	4,067
Female	4.7	3.7, 6.0	Reference		3,940
**Age (years)**					
10–12	4.2	3.1, 5.6	Reference	-	2,634
13–15	6.4	3.9, 10.5	1.5 (1.1, 2.0)	0.004	2,561
16–18	5.0	3.8, 6.5	1.0 (0.7, 1.3)	0.961	2,812
**Currently in school**					
No	6.1	4.2, 8.8	Reference		1,305
Yes	4.9	3.7, 6.5	0.6 (0.5, 0.8)	< 0.001	6,702
**Area of residence**					
Rural	4.2	3.4, 5.3	Reference	-	4,451
Urban	7.9	5.0, 12.3	1.4 (1.1, 1.8)	0.013	3,556
**Household Characteristics**					
**Religion**					
Hindu	5.3	4.0, 6.9	Reference		5,646
Muslim	4.9	3.4, 7.1	0.8 (0.6, 1.2)	0.288	974
Christian	4.0	1.9, 8.1	0.6 (0.3, 1.4)	0.268	856
Sikh	0.5	0.1, 2.1	0.1 (0.0, 1.1)	0.061	185
Other	9.8	3.3, 25.7	1.7 (0.7, 3.9)	0.221	346
**Caste/Tribe**					
Scheduled Caste/ Scheduled Tribe	4.8	3.5, 6.4	Reference		3,030
Other Backward Class	5.3	3.4, 8.3	1.0 (0.7, 1.3)	0.843	2,614
Other	5.6	4.1, 7.5	1.1 (0.8, 1.4)	0.769	2,363
**Wealth Index**					
Poorest	2.4	1.4, 4.1	Reference		606
Poor	4.9	3.2, 7.4	2.1 (1.3, 3.3)	0.001	1,035
Middle	3.9	2.5, 6.0	1.7 (1.1, 2.7)	0.029	1,604
Rich	6.1	4.3, 8.6	2.6 (1.6, 4.1)	< 0.001	2,200
Richest	8.3	4.7, 14.1	3.4 (2.1, 5.5)	< 0.001	2,562
**Unhealthy Diet **^**a**^					
No	5.1	4.0, 6.5	Reference		7,242
Yes	6.6	3.2, 13.0	1.1 (0.7, 1.6)	0.657	765

NCEP ATP III criteria was used for metabolic syndrome

^a^Daily consumption of either fried foods (poori, pakora, vada, samosa, tikki etc.) or junk foods (burger, pizza, pasta, instant noodles) or sweets (Indian sweets, pastries/cakes, donuts) or aerated drinks. Adjusted for sex, age, current schooling status of adolescents, area of residence, religion, caste, and household wealth

**Fig. 2:**
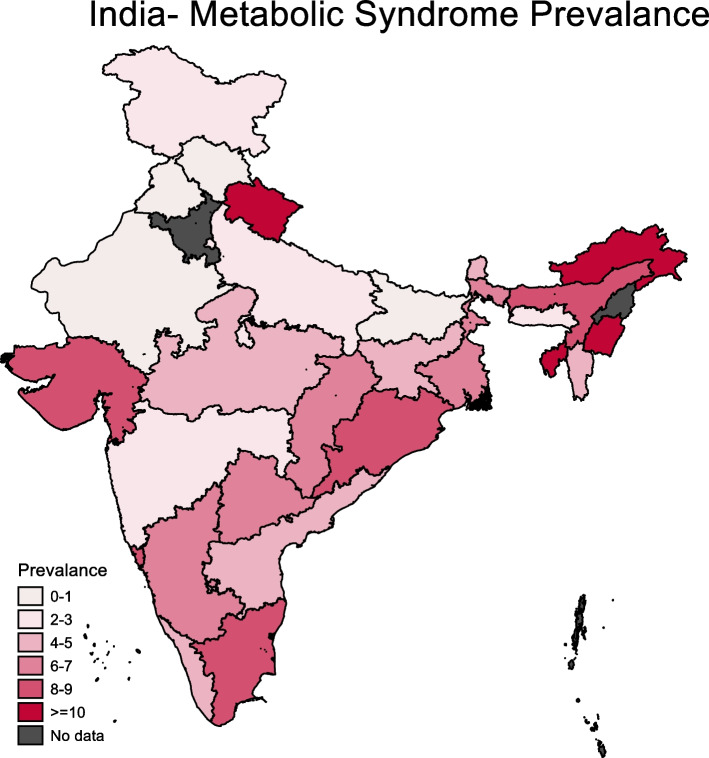
Prevalence of metabolic syndrome among adolescents by state in India, CNNS 2016–18. NCEP ATP III criteria modified for age was used for metabolic syndrome. Results for Haryana and Nagaland are not presented as the sample size was < 50

Wide interstate variability was observed in the prevalence of MS, ranging from less than 1% in Punjab to as high as 17% in Manipur (Fig. 2). In 15 states, the prevalence of MS was higher than the national-level prevalence. Further, in four states—Uttarakhand, Arunachal Pradesh, Manipur and Tripura—the prevalence was higher than 10%. In terms of the burden of MS, this study estimated 14.2 million adolescents (range 11.2 million–17·9 million) aged 10–19 years as having MS in 2017 in India (Table 5). Together, only six states (Uttar Pradesh, Karnataka, Gujarat, Tamil Nadu, Madhya Pradesh and West Bengal) contributed to 53% of MS burden in the country.

**Table 5:** Medium, low and high estimates of total number of adolescents with metabolic syndrome, by state in India in 2017

**State**	**Prevalence (CI)**	**Burden**		
		**Medium**	**Low**	**High**
Andhra Pradesh	4.4 (1.8–10.6)	409,174	166,632	981,277
Arunachal Pradesh	11.2 (7.1–17.3)	44,956	28,474	69,380
Assam	8.1 (3.4–17.9)	563,796	236,948	1,247,460
Bihar	1.8 (1–3.3)	495,024	273,494	902,530
Chhattisgarh	6.3 (3.9–9.9)	386,478	240,777	611,203
Goa	8.2 (5.3–12.5)	18,242	11,776	27,774
Gujarat	8.1 (4.7–13.7)	1,037,415	601,957	1,754,640
Haryana	(-)	0	0	0
Himachal Pradesh	1.9 (0.3–11.5)	23,757	3751	143,795
Jammu & Kashmir	2.1 (1–5)	58,700	27,430	137,150
Jharkhand	4.4 (1.8–10.7)	363,363	148,311	881,629
Karnataka	7.4 (4.9–11.1)	854,292	567,213	1,284,910
Kerala	5.9 (3.2–10.7)	349,771	190,028	635,407
Madhya Pradesh	4.8 (1.7–13)	860,693	303,564	2,321,370
Maharashtra	3.3 (1.6–6.4)	707,940	346,393	1,385,570
Manipur	16.5 (11.1–23.8)	111,464	75,121	161,071
Meghalaya	3.3 (1.3–8)	26,770	10,578	65,093
Mizoram	4.8 (2–11.1)	11,626	4814	26,718
Nagaland	(-)	0	0	0
NCT of Delhi	3.4 (1.4–8.4)	123,304	50,328	301,969
Odisha	8.4 (5.5–12.7)	722,895	472,200	1,090,353
Punjab	0.5 (0.1–2)	25,458	5417	108,332
Rajasthan	1.7 (0.4–6.6)	298,785	71,565	1,180,825
Sikkim	5.9 (3.7–9.4)	8600	5375	13,655
Tamil Nadu	9.1 (4.8–16.7)	1,151,024	605,138	2,105,377
Telangana	7.5 (4.6–12)	531,673	327,842	855,239
Tripura	12.3 (6.9–20.8)	86,684	48,786	147,066
Uttar Pradesh	2.2 (0.6–7.5)	1,244,017	342,390	4,279,875
Uttarakhand	10 (3.7–24.1)	243,027	90,372	588,638
West Bengal	6.5 (4.3–9.8)	1,236,328	812,876	1,852,602
**India**	**5.2 (4.1–6.5)**	**14,268,398**	**11,293,520**	**17,904,361**

Population projection for 10–19 age group using Census 2001 and 2011 data

Population projection Year 2017 (using decadal GR = ([population 2011-population 2001)/population 2001]*time)*population 2011 + population 2011

## Discussion

MS is characterised by a set of conditions associated with metabolic disorders and can be referred to as insulin resistance syndrome, dysmetabolic syndrome, syndrome X, Reaven syndrome, metabolic cardiovascular syndrome, to name a few. Several definitions of MS have been used across the globe; however, dyslipidemia (hypertriglyceridemia and low levels of high-density lipoprotein cholesterol), elevated blood pressure, impaired glucose tolerance and central adiposity are common to all. The prevalence of MS varies depending on the diagnostic criteria used. This study used the NCEP ATP III criteria modified for age and observed a prevalence of 5.2% among adolescents aged 10–19 years. Recent studies in India also reported clustering of MS risk factors among adolescents and reported similar prevalence ranging from 3.0% to 6.5% [4, 25–28].

The present study showed a higher prevalence of MS in males (5.7%) than females (4.7%). Though these results were concurrent with some previous studies, they conflicted with the findings of others. For example, in the study undertaken by Singh et al.in the state of Jammu and Kashmir, a significantly higher prevalence among males was reported, whereas two other studies undertaken in north India reported either no such difference or a higher prevalence among females [4, 25, 28]. The reason for these differences could be the small sample size in those studies or because different methodology was used to recruit study participants. For example, in the study conducted by Kapil et al.in the national capital territory of Delhi, participants were selected from schools that catered to high income group whereas we recruited a nationally representative sample using a multi-stage, stratified, probability proportion to size cluster sampling method. Further, the reasons could also be because they were undertaken in specific states and contextual state-level factors might have contributed to those differentials.

As per the findings of this study, adolescents who resided in urban areas had higher odds of having MS as compared to those living in rural areas (7.9%vs 4.2%). There are conflicting observations reported in prior research, with some studies reporting findings that are similar to what was observed in the present study and other studies reporting no such rural–urban differentials [4, 34]. The probable reason for observing higher prevalence in adolescents living in urban areas could be because of changes in the standards of living, unhealthy dietary habits and adoption of sedentary lifestyles. Several studies conducted in India have highlighted this point regarding the sedentary lifestyle of urban India adolescents and how this may be affecting their health [35]. With growing income in urban India, increase in the ownership of television and computer, it is possible that the adolescents are more and more engaging in sedentary lifestyle. Studies have also reported that low physical activity is associated with increased risk of MS [36]. Similarly, adolescents belonging to households from higher wealth quintiles had higher odds of having MS as compared to those from poorer households, which is in confirmation with studies conducted across India, where they found higher prevalence of overweight adolescents in affluent households [37]. Probable reason could be increased access to high-fat diet or junk food.

The most common component of MS observed in this study was low HDL-cholesterol, which is similar to what was reported in several previous studies undertaken in India and elsewhere [4, 38]. A very small percent of adolescents in this study had 4 to 5 risk factors of MS (0.5%), which is in contrast to an earlier study conducted in India where no adolescent was reported as having four or five components of MS [4], probably due to the small sample size.

This study observed a wide variability in the prevalence of MS across states and in 15 states the prevalence was higher than the national average. It was not possible to compare this with previous studies due to lack of studies reporting state level prevalence for all the states. However, looking at the trends related to overweight and obesity in previous research work in India, the probable reason for this wide geographic variability could be due to multiple factors, such as differences in lifestyles, the level of urbanisation, access to fast food outlets, etc.. In terms of number of adolescents, six states (Uttar Pradesh, Karnataka, Gujarat, Tamil Nadu, Madhya Pradesh and West Bengal) contributed to more than half the burden of MS in the country.

This study had several limitations. First, there is little consensus on the definition to be used for MS. The NCEP ATP III definition for MS was used in this study and the prevalence might change if a different definition was used to measure associated conditions of MS. Second, the fasting status considered in the study was based on self-reported information and the limitation of self-reported data is widely recognized. Similarly, some of the socio-demographic variables included in this study such as the items owned by the household, which was used to derive household wealth variables, were based on self-reported responses. Finally, the findings of this study are limited to adolescents and therefore cannot be generalized to the entire population, including all the age groups. Nonetheless, these limitations do not compromise the findings of this study and to the knowledge of the research team of this study, this is the first attempt made to estimate the prevalence and distribution of MS based on a nationally representative sample of adolescents in India. Future research using various other definitions of MS will help gather a range of estimates. Further, contextual state-level factors need to be explored to better understand the geographical variations in MS, as observed by this study, to design targeted interventions promoting lifestyle changes among adolescents to prevent disease progression into adulthood.

## Conclusion

In conclusion, the findings of this study highlight MS as a growing threat to the health of people in India. It is still clustered in urban areas, among economically better off households and in certain states. There is an increasing urgency to focus on early screening for metabolic abnormalities, especially in high prevalence/burden areas and promote healthy lifestyles among adolescents, given the risk that adolescents diagnosed with MS could develop chronic diseases in adulthood if MS goes unaddressed in early stages.

## Data Availability

Reasonable request for data used in this article may be made to the corresponding author.

## Abbreviations

AACE: American Association of Clinical Endocrinologists
AIIMS: All India Institute of Medical Sciences
AOR: Adjusted odds ratios
BMI: Body mass index
CDSA: Clinical Development Services Agency
CNNS: The Comprehensive National Nutrition Survey
CI: Confidence Intervals
GoI: Government of India
DBP: Diastolic blood pressure
HDL cholesterol: High-density lipoprotein cholesterol
SBP: Systolic blood pressure
MoHFW: Ministry of Health and Family Welfare
MS: Metabolic Syndrome
NCEP-ATP III: National Cholesterol Education Program and Adult Treatment Panel III
NFHS: National Family Health Survey
NHANES: National Health and Nutrition Examination Survey
NIN: National Institute of Nutrition
PGIMER: Postgraduate Institute for Medical Education and Research
QC: Quality Control
SD: Standard Deviation
TAG: Technical Advisory Group
WHO: The World Health Organization
WC: Waist circumference
